# Corrigendum

**DOI:** 10.1002/hsr2.848

**Published:** 2022-09-26

**Authors:** Robabeh Abedini, Alireza Abdshah, Narges Ghandi, Atefe Janatalipour, Sara Torabi, Maryam Nasimi

**Affiliations:** ^1^ Department of Dermatology Razi Hospital Tehran University of Medical Sciences Tehran Iran

This article serve as an erratum to the publication “Areata under treatment with Diphenylcyclopropenone (HSR‐2021‐06‐0515.R1).”

In the original article, we mentioned that treatment satisfaction Questionnaire for Medication (TSQM) has four subscales which are scored out of 100, and then added to calculate a final score out of 400. Per the guideline of the organization, the TSQM was designed and validated originally to be reported as four separate scores only. It was not investigated this way.

We reported the aforementioned four distinct scores, which is in line with the TSQM manual, then we added them to get a final score out of 400. After the discussion with members of the IQVIA, we were informed that while those four scores are correct, we should mention that the scores from 400 should be omitted. As the total score was only one of the outcomes we investigated, this error does not change the outcome of our study, the conclusions, or the recommendations. We have investigated, in detail, the associations between the four individual scores and the independent variables, and merely used the overall score as an extra measure. While none of the other parameters in our study are affected significantly, and none of the conclusions change, the authors regret this error.

